# Dual trajectories of antiretroviral therapy adherence and polypharmacy in women with HIV in the United States

**DOI:** 10.1186/s12981-023-00520-4

**Published:** 2023-05-13

**Authors:** Abubaker Ibrahim Elbur, Musie Ghebremichael, Deborah Konkle-Parker, Deborah L Jones, Shelby Collins, Adaora A. Adimora, Michael F. Schneider, Mardge H. Cohen, Bani Tamraz, Michael Plankey, Tracey Wilson, Adebola Adedimeji, Jessica E. Haberer, Denise L. Jacobson

**Affiliations:** 1 Center for Global Health, Massachusetts General Hospital, Boston, MA USA; 2 The Ragon Institute of MGH, MIT, and Harvard, Cambridge, MA USA; 3 Schools of Nursing, Medicine and Population Health, University of Mississippi Medical Center, Jackson, MS USA; 4 Department of Psychiatry & Behavioral Sciences, University of Miami Miller School of Medicine, Miami, FL USA; 5 Division of Infectious Disease, Emory University School of Medicine, Atlanta, GA USA; 6 Department of Medicine, University of North Carolina at Chapel Hill, Chapel Hill, NC USA; 7 Department of Epidemiology, Johns Hopkins Bloomberg School of Public Health, Baltimore, USA; 8 Department of Medicine, Stroger Hospital of Cook County, Chicago, IL USA; 9 School of Pharmacy, University of California, San Francisco, San Francisco, CA USA; 10 Department of Medicine, Division of General Internal Medicine, Georgetown University Medical Center, Washington, DC USA; 11 School of Public Health, SUNY Downstate Health Sciences University, Brooklyn, NY USA; 12 Dept of Epidemiology and Population Health, Albert Einstein College of Medicine, Bronx, NY USA; 13 Center for Global Health, Massachusetts General Hospital, Harvard Medical School, Boston, MA USA; 14 Center for Biostatistics in AIDS Research, Department of Biostatistics , Harvard T. H. Chan School of Public Health, Boston, MA USA

## Abstract

**Background:**

Polypharmacy, using five or more medications, may increase the risk of nonadherence to prescribed treatment. We aimed to identify the interrelationship between trajectories of adherence to antiretroviral therapy (ART) and polypharmacy.

**Methods:**

We included women with HIV (aged ≥ 18) enrolled in the Women’s Interagency HIV Study in the United States from 2014 to 2019. We used group-based trajectory modeling (GBTM) to identify trajectories of adherence to ART and polypharmacy and the dual GBTM to identify the interrelationship between adherence and polypharmacy.

**Results:**

Overall, 1,538 were eligible (median age of 49 years). GBTM analysis revealed five latent trajectories of adherence with 42% of women grouped in the consistently moderate trajectory. GBTM identified four polypharmacy trajectories with 45% categorized in the consistently low group.

**Conclusions:**

The joint model did not reveal any interrelationship between ART adherence and polypharmacy trajectories. Future research should consider examining the interrelationship between both variables using objective measures of adherence.

**Supplementary Information:**

The online version contains supplementary material available at 10.1186/s12981-023-00520-4.

## Introduction

In the United States, women constitute a significant minority of the HIV epidemic. According to the Centers for Disease Control and Prevention (CDC) statistics, in 2020, women constituted 18% of the new cases diagnosed with HIV in the United States [1].

Adherence to antiretroviral therapy (ART) is a key component of successful treatment, yet women’s ART adherence is suboptimal. For example, in a recent analysis of 2,601 women enrolled in the Women’s Interagency HIV Study (WIHS) in the United States, 18% of participants reported taking ART less than 75% of the time, and only 52% were virally suppressed [2]. Substantial advances have been made in developing potent antiretroviral drugs with lower toxicity and improved pharmacokinetic profiles [3].

Concurrent with challenges in adherence are challenges that arise with increasing rates of premature comorbid conditions that commonly arise as people with HIV (PWH) age. Women followed in the WIHS were found to have a significantly higher mean number of non-AIDS-related comorbidities compared to HIV seronegative women (3.6 vs. 3.0, respectively) [4]. An increase in comorbidities typically leads to polypharmacy, defined as the concomitant use of five or more non-HIV medications with ART [5]. Polypharmacy can be associated with negative consequences such as nonadherence to treatment due to pill burden, increased medication side effects, and drug-drug interactions [6]. Among women with HIV (WHIV) and without HIV enrolled in WIHS both polypharmacy and the use of neurocognitive adverse effects medications were found to be strong determinants of falls [7]. Another study among elderly WHIV (> 50 years) in WIHS revealed a strong association between polypharmacy and poor executive function and processing speed [8]. In addition, polypharmacy can cause physical decline, hospitalization, and death [6]. Furthermore, polypharmacy can have a negative impact on the health-related quality of life outcomes of PWH [9].

A few studies have examined the impact of polypharmacy on adherence to ART therapy [10, 11]. For example, Zheng et al. [10] found that the high medication burden caused by polypharmacy, as measured by the Living with Medicines Questionnaire (LMQ), had a negative impact on ART self-reported adherence scores in a cross-sectional study of 185 Chinese PWH aged 50 years and above.

Static measures are commonly used to explore both adherence and polypharmacy (e.g., cross-sectional assessments of the proportion of days covered to measure adherence and *pill count*); however, these approaches fall short of capturing the dynamic nature of long-term behaviors and phenomena given the changing nature of many influencing factors [12]. Group-based trajectory modeling (GBTM) is a novel statistical data-driven approach used for analyzing developmental trajectories (i.e., the evolution of an outcome over age or time) [13]. In most studies, GBTM defines four to six trajectory groups that describe the dynamic behavior of longitudinal adherence [13]. Identifying individuals with suboptimal adherence trajectories is valuable because it informs the design of tailored interventions based on the group’s specific characteristics [14]. The identification of multiple characteristics defining the group may be more relevant for screening purposes when compared to single risk factors. Moreover, compared to the conventional method of determining the level of adherence based on dichotomized cut-offs and specific time points, GMBT can predict treatment outcomes with trends over time, both from initiation and during chronic care [15]”.

To the best of our knowledge, no other study has used dual GBTM to delineate the evolution of adherence and polypharmacy over time and characterize the interrelationships between them. We hypothesized that GBTM analysis would delineate different latent trajectories of ART adherence and of polypharmacy with variation in the predictors of group membership among trajectories of both variables; we specifically anticipated that a high polypharmacy trajectory would be associated with low ART adherence among WHIV enrolled in WIHS.

## Methods

### Study design

We conducted a retrospective analysis of longitudinal data of WHIV enrolled in WIHS between April 2014 to September 2019. WIHS is the oldest and largest prospective cohort of women living with or at risk for HIV [16, 17], which has now been combined with the Multicenter AIDS Cohort Study (MACS) to form the MACS/WIHS Combined Cohort Study (MWCCS). The main goal of WIHS is to investigate the natural history of HIV treatment and prevention in women in the United States. WIHS was founded in 1993, and women were recruited in four waves (1994-95, 2001–2002, 2011–2012, and 2013–2015). During the first three waves, participants were enrolled from the Bronx and Brooklyn, New York; Washington, DC; Los Angeles and San Francisco, California; and Chicago, Illinois. During the fourth wave, more participants were recruited from other research sites in Atlanta, Georgia; Chapel Hill, North Carolina; Miami, Florida; Birmingham, Alabama; and Jackson, Mississippi [18]. The Institutional Review Board approved this study at each of the study sites.

### WIHS data

At semi-annual study visits, data collection involved clinical exams, blood sample collection, and interviewer-administered questionnaires to collect basic sociodemographic, behavioral, and clinical data. This analysis included data on age (years), geographical region (Midwest, South, West, and Northeast), race (non-Hispanic white, non-Hispanic African American, and Hispanic of any race), educational level (below secondary, completed secondary, some college/completed college), household income (categorized here as <$24,000 vs. ≥$24,000), employment status (employed vs., unemployed), time since diagnosis with HIV (years), smoking status (women who never smoked, women who smoke, women who previously smoked), alcohol intake (abstainer, > 0–7 drinks/week, > 7–12 drinks/week, > 12 drinks/week), substance use at baseline (marijuana or hash, crack, cocaine, heroin, illicit methadone, methamphetamines, amphetamines, narcotics, hallucinogens, other drugs), and depression status (measured by the Center for Epidemiological Studies Depression (CES-D) Scale with a score of ≥ 16 indicating the presence of depressive symptoms and < 16 indicating no depression) [19]. It also included self-reported non-HIV and HIV medication use and adherence to ART. Self-reported ART adherence over the past month is categorized as “100% of the time”, “95–99% of the time”, “75–94% of the time”, “<75% of the time,” and “I have not taken any of my prescribed medications.”

### Study participants

We included WHIV enrolled in WIHS between April 2014 to September 2019 aged ≥ 18 years on ART, who had at least three self-reported adherence measurements and three visits with recorded data on non-HIV medications. Women with less than three adherence measurements and less than three visits with non-HIV medications recorded data were excluded due to the prerequisites for fitting GBTM. The first adherence visit between the above dates was designated as the “baseline” visit.

### Data analysis

#### Polypharmacy

To assess polypharmacy, we determined the number of non-HIV medications being taken at the time of each visit, including all reported prescribed and over-the-counter medications, herbal supplements, topical and ophthalmic, and as-needed medications. If a medication contained two or more pharmacologically active agents, each substance was counted individually in the analysis. Multivitamins that have multiple ingredients were counted as one. Herbals were counted as one regardless of the mixture. At each visit, polypharmacy was defined as the concomitant use of five or more non-HIV medications [6], and no polypharmacy was defined as zero to four non-HIV medications. Prescription-only polypharmacy was defined as five or more prescription-only medications, and no prescription polypharmacy was defined as zero to four non-HIV medications, excluding over-the-counter medications, herbal supplements, and as-needed medications. We defined polypharmacy as 5 or more medications because this threshold has been associated with poor treatment outcomes (e.g., disability, falls, frailty, and death) [20]. Furthermore, the definition has been used in most studies conducted among PWHIV to assess polypharmacy [21]. We assessed the pharmacologically active ingredients rather than the number of pills because the former is more comprehensive and can capture whether nonadherence to ART is due to pill burden or drug unintended effects.

#### Group-based trajectory modeling

We used GBTM to identify latent adherence and polypharmacy groups using a censored normal model and a logit model, respectively [22]. Each of the following steps was performed to identify the number and shape of trajectory groups for adherence and polypharmacy separately. Initially, the analysis procedure involved fitting several models sequentially to determine the appropriate number of trajectory groups. The second step entailed visual inspections and determining trajectory shapes considering constant, linear, quadratic, and cubic specifications. A set of criteria was considered to determine model fit namely, (1) Bayesian Information Criteria (BIC) with smaller values indicating better model fit, (2) the mean posterior probability of membership within each group (entropy) with values > 0.70 generally indicating acceptable classification, (3) the smallest group with at least 5% of the sample, (4) a tight confidence interval around estimated group membership probabilities and statistically significant groups (*P* < 0.05), and (5) parsimony in the model with few classes and parameters probabilities [13]. In addition, the model selection process was based on subject matter knowledge about the patterns of both variables and the interpretability of the model. The final step involved estimating a dual trajectory model using the univariate models that had been identified. We tested several models by varying the number of groups in each variable to ensure that the groups identified in univariate GBTM analysis in both adherence and polypharmacy were the best models for the joint analysis. The joint model summarized the interrelationships between adherence and polypharmacy trajectories as conditional probabilities of each variable on the other and their joint probabilities as well [13]. We assumed that the missingness was fully random, in which case GBTM would account for the missingness by fitting the model with maximum likelihood estimation and giving asymptotically unbiased parameter estimates [13].

#### Descriptive and comparative analysis

For each trajectory within adherence and polypharmacy, categorical variables were presented as numbers, and percentages and continuous variables were summarized as median and interquartile ranges (IQR). Comparisons were made across trajectories of adherence using the Chi-square test. We measured the association between the percentage of women who reported adherence at a level of ≥ 95% and the percentage of women on polypharmacy during the study period using the Pearson correlation method.

#### Predictors for membership in adherence trajectories

We fitted multinomial logistic regression analysis to identify predictors of the group membership of adherence, namely, age (years), race, educational level, annual income, alcohol intake, history of smoking, cumulative years in ART, depression, and substance use. We planned to use the group with the highest level of adherence probability as a reference group in the model. As an initial step, we performed univariable analyses, and covariates with P-values < 0.25 were selected to be included in the final model.

#### Sensitivity analyses

In a sensitivity analysis, we used the above-mentioned definition of prescription-only polypharmacy to classify women as having polypharmacy or not at every study visit. We assumed that the state of polypharmacy using prescription-only medications potentially affects ART adherence more than polypharmacy of a combination of prescription-only, over-the-counter, as-needed medications, and herbal supplements. We used GBTM to identify polypharmacy trajectories using the logit model, following the same statistical steps and criteria for identifying group numbers and trajectory shapes described above. Furthermore, we used GBTM to conduct another sensitivity analysis in which we considered the number of non-HIV medications as a continuous variable. We included all prescription-only, over-the-counter, as-needed medications, and herbal supplements in this analysis. We assumed that using a large number of non-HIV medications concurrently with ART at any given time would create a burden and, as a result, influence adherence. Using the same statistical procedure described above, we used the censored normal model to identify the number and shapes of trajectories.

Data analysis was conducted using Stata version 16, and the Stata Plugin was used to estimate GBTM parameters.

## Results

### Participants’ baseline characteristics

Overall, 1,678 women were followed during the study period, of whom 1,538 (91.7%) were eligible for this analysis, contributing to 14,080 participant -visits. Self-reported ART adherence was recorded in 13,692 (97.2%) participant-visits and use of non-HIV medications in 13,987 (99.0%) participant-visits. The participants’ median age was 49 (interquartile range [IQR] 42–54) years and the majority (1,117; 72.6%) were non-Hispanic African Americans.

### Group-based trajectory modeling

#### Average trend and association of adherence and polypharmacy over the study period

**Fig. 1:**
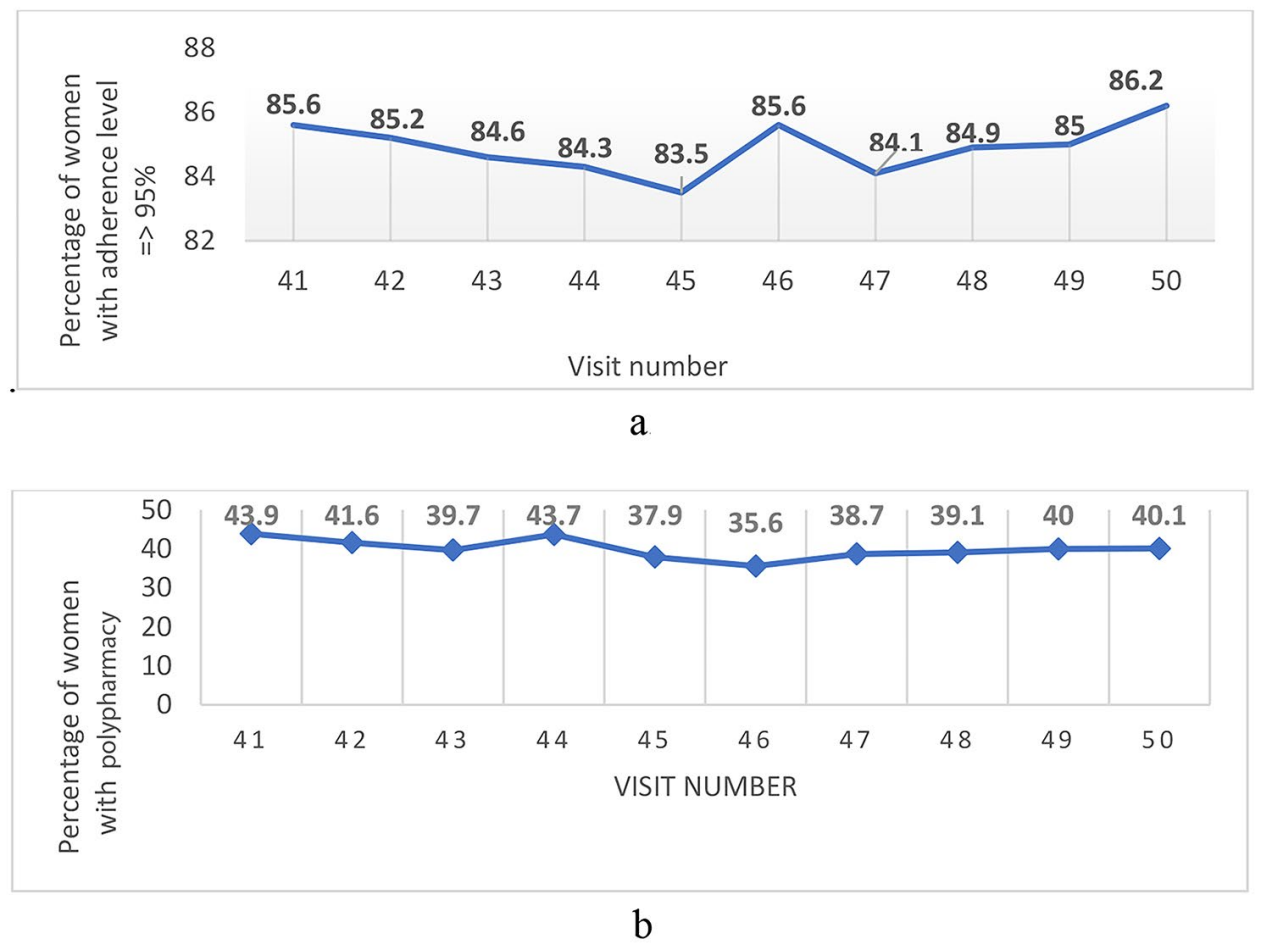
**a** Trends of > 95% self-report adherence to ART over the study period. **b** Trends of polypharmacy (5 + medications) over the study period

Figure 1a and b show the trends in the percentages of women with adherence levels ≥ 95% and the percentages of women with polypharmacy at each study visit. There were no linear trends in adherence or polypharmacy over the study period, with only small fluctuations in percentages between study visits in both variables. The overall correlation between adherence and polypharmacy during the study period was 0.11; (*P* = 0.75).

#### Trajectories of ART adherence

**Fig. 2:**
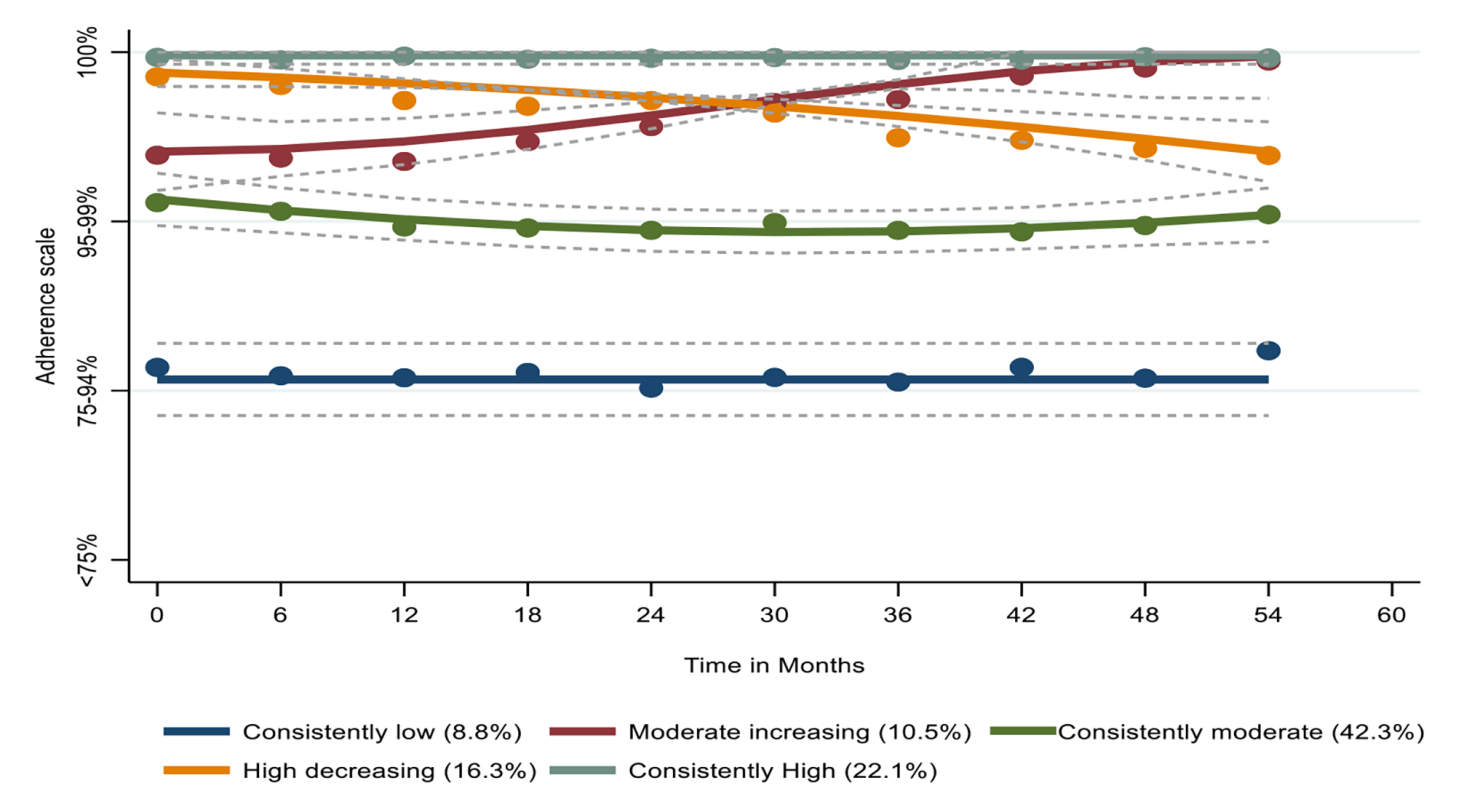
Trajectories of adherence to antiretroviral therapy

We identified five latent trajectories of adherence, namely ‘consistently low’ (N = 136; 8.8%), ‘consistently moderate’ (N = 650; 42.3%), ‘moderate increasing’ (N = 162; 10.5%) ‘high decreasing’ (N = 250; 16.3%), and ‘consistently high’ (N = 340; 22.1%), as depicted in Fig. 2. Women who belonged to the consistently low adherence group were more likely to be younger, recruited from study sites in western U.S, African American, highly educated (had Some college or completed college), used alcohol in different quantities, women who smoke, and experience depression symptoms at baseline compared to women in other groups. Table 1 shows women’s characteristics at the start of the current study by adherence trajectories. We compared the baseline characteristics of the women in the high decreasing and moderate increasing groups because their adherence patterns changed over time while the other three groups remained relatively stable. The women who followed the high decreasing trajectory were more likely to consume high quantities of alcohol compared to women who were members of the moderate increasing group, as shown in Table 2.

**Table 1:** Women’s characteristics at the start of the current study by adherence trajectories in the WIHS cohort

Characteristic		Adherence trajectories					
	Consistently high (n = 340)	High decreasing (n = 250)	Consistently moderate (n = 650)	Moderate increasing (n = 162)	Consistently low (n = 136)	Total (n = 1538)	P-value
Geographical region							
Midwest West South Northeast	33 (17.6) 25 (13.4) 143 (25.1) 139 (23.3)	21(11.2) 21(11.3) 86 (15.1) 122 (20.5)	100 (53.5) 87 (46.8) 238 (41.8) 225 (37.7)	20(10.7) 27(14.5) 54 (9.4) 61(10.2)	13(7.0) 26 (14.0) 48(8.4) 49(9.2)	187 (12.2) 186 (12.1) 569 (37.0) 596 (38.7)	**< 0.001**
Age groups							
< 50 years ≥ 50 years	166 (48.8) 174 (51.2)	134 (53.6) 116 (46.4)	358 (55.1) 292 (44.9)	79 (48.8) 83 (51.2)	93 (68.4) 43 (31.6)	830 (54.0) 708 (46.0)	**0.002**
Race							
White African American Hispanic Others	35 (10.3) 244 (71.8) 54 (15.8) 7 (2.1)	31 (12.4) 176 (70.4) 36 (14.4) 7 (2.8)	72 (11.1) 483 (74.3) 74 (11.4) 21 (3.2)	13 (8.0) 107 (66.0) 37 (22.8) 5 (3.1)	10 (7.4) 107 (78.7) 14 (10.3) 5 (3.6)	161(10.5) 1117 (72.6) 215 (14.0) 45 (2.9)	**0.04**
Education							
Below secondary school Completed secondary school Some /completed college	127 (37.4) 118 (34.7) 95 (27.9)	83 (33.3) 78 (31.3) 88 (35.3)	186 (28.7) 215 (33.1) 248 (38.2)	60 (37.0) 48 (29.6) 54 (33.3)	48 (35.5) 34 (25.2) 53 (39.3)	504 (32.8) 493 (32.1) 538 (35.1)	**0.024**
Employment status							
No Yes	225 (66.2) 115 (33.8)	172 (68.8) 78 (31.2)	416 (64.1) 233 (35.9)	108 (67.1) 53 (32.9)	84 (62.2) 51 (37.8)	1005 (65.5) 530 (34.5)	0.61
Annual income							
≤ $24,000 >$24,000	270 (79.4) 70 (20.6)	197 (78.8) 53 (21.2)	478 (73.8) 170 (26.2)	123 (75.9) 39 (24.1)	106 (77.9) 30 (22.1)	1174 (76.4) 362 (23.6)	0.26
Alcohol categories (%)							
0 drinks/week >0–7 drinks/week >7–12 drinks/week >12 drinks/week	224 (65.9) 89 (26.2) 13 (3.8) 14 (4.1)	125 (50.4) 98 (39.5) 8 (3.2) 17 (6.8)	340 (52.3) 233 (35.8) 32 (4.9) 45 (6.9)	105 (64.8) 49 (30.2) 3 (1.8) 5 (3.1)	50 (36.8) 55 (40.4) 13 (9.6) 18 (13.2)	844 (54.9) 524 (34.1) 69 (4.5) 99 (6.4)	**< 0.001**
History of smoking status (%)							
Women who never smoked Women who smoke Women who previously smoked	130 (38.2) 113 (33.2) 97 (28.5)	78 (31.2) 98 (39.2) 74 (29.6)	235 (36.1) 239 (36.8) 176 (27.1)	51 (31.5) 61 (37.7) 50 (30.7)	38 (27.9) 69 (50.7) 29 (21.3)	532 (34.6) 580 (37.7) 426 (27.7)	**0.04**
Substance use							
Yes No	122 (35.9) 218 (64.1)	70 (28) 180 (72)	190 (29.4) 456 (70.6)	47 (29.0) 115 (71.0)	34 (25.2) 101 (74.8)	463 (30.2) 1070 (69.8)	0.1
Depression symptoms (%)							
No Yes	273 (80.3) 67 (19.7)	183 (73.2) 67 (26.8)	418 (64.3) 232 (35.7)	114 (70.4) 48 (29.6)	73 (53.7) 63 (46.3)	1061(69.0) 477 (31.0)	**< 0.001**

**Table 2:** Characteristics of women in moderate increasing and high decreasing adherence groups at baseline

Characteristic	Adherence trajectory		Total n (%) N = 412	P-value
	Moderate increasing n (%) N = 162	High decreasing n (%) N = 250		
Age groups				
< 50 years ≥ 50 years	79 (48.8) 83 (51.2)	134 (53.6) 116 (46.4)	213 (51.7) 199 (48.3)	0.33
Race				
White African American Hispanic Others	13 (8.0) 107 (66.0) 37 (22.8) 5 (3.1)	31 (12.4) 176 (70.4) 36 (14.4) 7 (2.8)	44 (10.7) 283 (68.7) 73 (17.7) 12 (2.9)	0.11
Education				
Below secondary Completed secondary Some college/ completed college	60 (37.0) 48 (29.6) 54 (33.3)	83 (33.3) 78 (31.3) 88 (35.3	143 (34.8) 126 (30.7) 142 (34.5)	0.74
Employment				
No Yes	108 (67.1) 53 (32.9)	172 (68.8) 78 (31.2)	280 (68.1) 131(31.9)	0.71
Annual income				
= < $ 24,000 >$ 24,000	123 (75.9) 39 (24.1)	197 (78.8) 53 (21.2)	320 (77.7) 92 (22.3)	0.49
Alcohol categories				
0 drinks/week >0–7 drinks/week >7–12 drinks/week >12 drinks/week	105 (64.8) 49 (30.2) 3 (1.9) 5 (3.1)	125 (50.4) 98 (39.5) 8 (3.2) 17 (6.9)	230 (56.1) 147 (35.8) 11(2.7) 22 (5.4)	**0.02**
History of smoking status				
Women who never smoked Women who smoke Women who previously smoked	51 (31.4) 61 (37.7) 50 (30.9)	78 (31.2) 98 (39.2) 74 (29.6)	129 (31.3) 159 (38.6) 124 (30.1)	0.94
Depression symptoms				
No Yes	114 (70.4) 48 (29.6)	183 (73.2) 67 (26.8)	297 (72.1) 115 (27.9)	0.53
Substance use				
No Yes	47 (29.0) 115 (71.0)	70 (28.0) 180 (72.0)	117 (28.4) 295 (71.6)	0.82

#### Trajectories of polypharmacy (considering all medications)

**Fig. 3:**
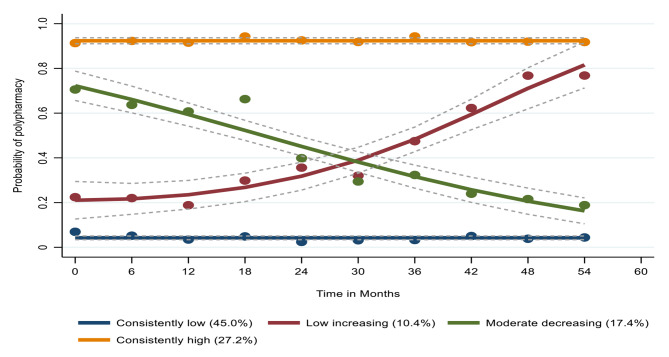
Trajectories of polypharmacy (considering all medications)

When considering all prescribed, over-the-counter, and as-needed medications and herbal supplements, GBTM identified four latent trajectories of polypharmacy, namely ‘consistently high’ (N = 418; 27.2%), ‘moderate decreasing’ (N = 268; 17.4%), ‘low increasing’ (N = 160; 10.4%), and ‘consistently low’ (N = 692; 45.0%), as shown in Fig. 3. The low increasing and moderate decreasing groups’ polypharmacy patterns altered over time, while the other groups remained largely steady. In comparing the baseline characteristics of the women who were members of those groups, we found that 11 (6.9%) of White women were in the low increasing group, while 31 (11.6%) were in the moderate decreasing group. In contrast, 125 (78.1%) African Americans were in the low increasing group, while 188 (70.1%) were in the moderate decreasing group, (*P* = 0.07**)**, as shown in supplemental Table 1.

#### Dual trajectory model

For the joint model, the number of groups identified in the univariate analysis for each variable was found to be optimal (Supplemental Table 2). The results showed small changes in the probabilities of group membership when comparing the univariate and joint models except in the consistently low and consistently moderate, as presented in Supplemental Table 3.

#### Interrelationships across the trajectory groups of adherence and polypharmacy (considering all medications)

Table 3a shows the probability of trajectory group membership in polypharmacy conditional on ART adherence. The results showed that half of the women in the moderate increasing adherence group were members of the consistently low polypharmacy group compared to 46%, 44%, and 41% of the women in the consistently moderate, consistently low, and consistently high adherence groups, respectively. Table 3b shows the probability of trajectory group membership in ART adherence conditional on polypharmacy groups. The analysis showed that across all polypharmacy groups, the consistently moderate adherence group constituted the largest component as follows: 42% of the consistently low, 40% of the consistently high, 35% of the moderate decreasing, and 34% of the low increasing polypharmacy groups. Table 3c reports the joint probability of the ART adherence and polypharmacy groups, which all sum to 1. As shown in Table 3c the joint probability for women to be members of both the high decreasing adherence group and low polypharmacy group was 18%, 11.0% for women who belonged to both the high decreasing adherence group and consistently high polypharmacy group, and 8% for women grouped in both the consistently high adherence and low polypharmacy group.

**Table 3:** Dual group-based trajectory modeling of polypharmacy and adherence to antiretroviral therapy

3a. Probability of polypharmacy group conditional on adherence group /all medications								
Adherence trajectory group	Polypharmacy trajectory group							
	Consistently high	Moderate decreasing		Low increasing		Consistently low	Total	
Consistently high	0.31	0.14		0.14		0.41*	1	
High decreasing	0.3	0.22		0.15		0.33	1	
Moderate increasing	0.33	0.15		0.02		0.5*	1	
Consistently moderate	0.28	0.16		0.10		0.46*	1	
Consistently low	0.13	0.28		0.15		0.44*	1	
3b. Probability of adherence conditional on polypharmacy/ all medications								
Adherence trajectory group	Polypharmacy trajectory group							
	Consistently high	Moderate decreasing		Low increasing		Consistently low		
Consistently high	0.22	0.15		0.23		0.19		
High decreasing	0.19	0.21		0.23		0.14		
Moderate increasing	0.12	0.08		0.02		0.11		
Consistently moderate	0.40*	0.35*		0.34*		0.42*		
Consistently low	0.07	0.21		0.18		0.14		
Total	1	1		1		1		
3c. Joint probability of adherence and polypharmacy / all medications								
Adherence trajectory group	Polypharmacy trajectory group							
	Consistently high		Moderate decreasing		Low increasing	Low		
Consistently high	0.06		0.03		0.03	**0.08**		
High decreasing	0.11*		0.06		0.04	0.18*		
Moderate increasing	0.05		0.04		0.03	0.06		
Consistently moderate	0.03		0.01		0.00	0.05		
Consistently low	0.02		0.04		0.02	0.06		

*Indicate high probability

#### Predictors of group membership in ART adherence trajectories

Table 4 shows the predictors of group membership for each ART adherence trajectory compared to the consistently high adherence group in the multivariable multinomial model. The results showed that significant predictors of being a member of the consistently low trajectory were younger age < 50 years, recruited from study sites in the western U.S, being non-Hispanic African American, higher education, any alcohol intake, being on ART for six or more years, and presence of depression symptoms at baseline.

**Table 4:** Predictors of group membership in ART adherence trajectories compared to the reference group consistently high adherence

Covariates	Consistently low (N = 136) aOR (95% CI)	Consistently moderate (N = 650) aOR (95% CI)	Moderate increasing (N = 162) aOR (95% CI)	High decreasing (N = 250) aOR (95% CI)
Geographical region				
Midwest West South Northeast	1.0 2.6 (1.1–6.3) * 0.8 (0.4–1.9) 1.1(0.5–2.3)	1.0 1.1(0.6- 2.0) 0.5 (0.3–0.9) * 0.6 (0.4-1.0) *	1.0 1.7 (0.8–3.7) 0.8 (0.4–1.7) 0.7 (0.4–1.3)	1.0 1.2 (0.5–2.7) 0.8 (0.4–1.6) 1.6 (0.9–2.9)
Age groups				
≥50 years < 50 years	1.0 2.8 (1.8–4.4) *	1.0 1.4 (1.1–1.9) *	1.0 1.2 (0.8–1.8)	1.0 1.4 (0.9-2.0)
Race				
White (Non-Hispanic) African American (Non-Hispanic) Hispanic Others	1.0 2.4 (1.1–5.5) * 1.6 (0.6–4.5) 2.7(0.7–11.1)	1.0 1.4 (0.8–2.3) 1.1 (0.6-2.0) 1.7 (0.7–4.7)	1.0 1.7 (0.9–3.5) 2.6 (1.2–5.9) * 2.1 (0.6-8.0)	1.0 0.9 (0.5–1.6) 0.9 (0.5–1.8) 1.1 (0.3–3.6)
Education				
Below secondary Completed secondary Some college/ completed college	1.0 1.1 (0.6–1.8) 2.1 (1.2–3.4) *	1.0 1.4 (0.9–1.9) 1.9 (1.3–2.7) *	1.0 1.0 (0.6–1.5) 1.4 (0.8–2.3)	1.0 1.1 (0.7–1.7) 1.6 (1.0-2.4) *
Annual income				
≤ $24,000 >$24,000	1.0 1.1 (0.6–1.8)	1.0 1.3 (0.9–1.8)	1.0 1.3 (0.8–2.1)	1.0 0.9 (0.6–1.4)
Alcohol categories				
0 drinks/week >0–7 drinks/week >7–12 drinks/week >12 drinks/week	1.0 2.5 (1.5-4.0) * 3.5 (1.4–8.4) * 4.6 (2.0-10.6) *	1.0 1.5 (1.1–2.1) * 1.4 (0.7–2.8) 2.0 (1.03–3.8) *	1.0 1.1 (0.7–1.8) 0.4 (0.1–1.6) 0.7 (0.2–2.1)	1.0 1.9 (1.3–2.8) * 1.0 (0.4–2.5) 2.1 (0.9–4.5)
History of smoking				
Women who never smoked Women who smoke Women who previously smoked	1.0 1.7 (0.9–3.2) 1.0 (0.5–1.9)	1.0 1.1 (0.7–1.6) 0.9 (0.6–1.4)	1.0 1.4 (0.8–2.4) 1.1 (0.6-2.0)	1.0 1.3 (0.8-2.0) 1.1 (0.6–1.8)
Cumulative years on ART				
< 6 years ≥ 6 years	1.0 2.0 (1.13.6) *	1.0 1.2 (0.8–1.8)	1.0 1.5 (0.9–2.5)	1.0 0.9 (0.6–1.4)
Depression symptoms No	1.0	1.0	1.0	1.0
Yes	3.8 (2.3–6.1) *	2.5 (1.9–3.6) *	1.8 (1.2–2.9) *	1.6 (1.1–2.4) *
Substance use at baseline				
No Yes	1.0 1.2 (0.7-2.0)	1.0 1.3 (0.9–1.9)	1.0 1.3 (0.8–2.1)	1.0 1.2 (0.8–1.9)

*P-value < 0.05

Abbreviation: aOR-adjusted odds ratio

#### Sensitivity analysis

The results of the sensitivity analysis are presented in the Supplemental file. Similar to the findings of the primary analysis, GBTM using prescription-only drugs and non-HIV drugs as a continuous variable identified four trajectory groups. As with the primary analysis, the joint model analysis in both cases revealed no interrelationship between adherence groups and polypharmacy considering prescription-only medication or non-HIV medications trajectory groups as a continuous variable.

## Discussion

Our analysis revealed five latent trajectories of adherence to ART, with the consistently moderate group accounting for the largest proportion of women (42%). Considering all non-ARV drugs, GBTM identified four polypharmacy categories, with 45% of the women following a low polypharmacy trajectory. The joint model did not reveal any apparent relationship between ART adherence and polypharmacy trajectories. Likewise, the sensitivity analyses that assessed (1) prescription-only medications and (2) non-HIV as a continuous variable did not identify any evidence of an interrelationship between both variables.

In the current analysis, GBTM provided a unique perspective on the evolving nature of WHIV adherence behavior, distinguishing five categories of adherence across time. Prior studies generally utilized a traditional method of categorizing adherence into two or, at most, three categories, which miss changes in adherence over time [23]. The experiences of women who followed the moderate increasing trajectory may seem to indicate minor changes in adherence (i.e., 95–99–100%); however, such a change may be clinically meaningful. That is, social desirability bias often results in over-reporting of adherence [24]. Any reported non-adherence is therefore likely accurate and the change to complete adherence may reflect significant improvement in pill taking and the corresponding clinical benefits. Additional qualitative research is needed to identify the factors that motivated and/or facilitated their improved adherence. These findings may help in designing interventions to improve adherence among similar women. In general, the findings revealed that most predictors of membership in the continuously low and consistently moderate adherence trajectories (i.e., younger age, being non-Hispanic African American, higher education, any alcohol intake, being on ART for six or more years, and depression) have been described in the literature [25]. However, classifying women based on their adherence trajectories may shed new light on the design of a focused intervention to target these factors to improve adherence. These results revealed that women who were members of the high decreasing trajectory were more likely to consume high quantities of alcohol compared to women who were members of the moderate increasing. In addition to having a direct impact on medication-taking behavior, alcohol consumption has a negative impact on the immune system, which could result in HIV progression [26]. In another study of nearly one thousand WHIV, excessive alcohol consumption was found to be a predictor of poor adherence to ART [27]. Women in the high decreasing may benefit from combined behavioral interventions that address both adherence and alcohol consumption, as such interventions have been shown to reduce alcohol consumption and improve treatment outcomes [28].

The use of GBTM as a novel statistical approach proved to be beneficial in the analysis of polypharmacy data. The model revealed four trajectories, with 45% of all women classified in the consistently low group, which means they never used more than five medications concurrently with ART. In comparison, Ware et al. [29] used GBTM to examine polypharmacy among men participating in the Multicenter AIDS Cohort Study (MACS); four groups were identified with 48% of participants falling into a non-polypharmacy group (less than five medications with ART). A notable difference was observed in the percentage of women who consistently remain in the high polypharmacy group compared to men in the MACS study (27.2% vs. 14.2%, respectively). A population-based study in Spain analyzed a large database (23,000 PWHIV) and found polypharmacy was more common among women than among men (45% vs. 30%, respectively) [30]. High polypharmacy among women may be associated with higher rates of chronic illnesses than their male counterparts [31]. Our results showed that 17.5% of the women with polypharmacy started at a moderate level at the beginning of the study and then decreased gradually during the follow-up period. The women in this group may have stopped the use of drugs for acute illnesses or perhaps vitamins and/or herbal supplements. On the contrary, nearly 10% began with a low level of polypharmacy and gradually increased during the study. The gradual increase could be attributed to the development of chronic comorbidities over time.

Contrary to our hypothesis, neither the primary nor sensitivity analyses revealed any interrelationship between adherence groups and polypharmacy groups. Cantudo-Cuenca et al. [32] examined the impact of polypharmacy on adherence to ART, as measured by pharmacy dispensing records and the Morisky Medication Adherence Scale (MMAS), in a cross-sectional study in Spain (n = 594) and found that polypharmacy reduced adherence to ART. The difference between the two studies could be attributed to the difference in the data analysis methods, the short duration of follow-up (12 months), the smaller sample size, and the methods used to measure adherence. A possible explanation for our results is that there may be a presence of an effect modifier/s between adherence and polypharmacy, for example, depression or age. This assumption will be thoroughly investigated in further research.

The findings of this analysis have the following clinical recommendations. Firstly, adherence to ART is dynamic, so healthcare providers should counsel women about the need for a lifelong commitment to ART, check to what extent each woman adheres to her medication, explore barriers if any, and provide the necessary education and support at every clinical encounter. Healthcare providers should pay particular attention to women who have the characteristics of those belonging to low, moderate, and moderate decreasing adherence trajectories. Secondly, although we did not observe an interrelationship between polypharmacy and ART adherence, further study is needed to explore potential associations using objective adherence measures. Moreover, polypharmacy is associated with inappropriate prescribing and has known negative effects, including drug-drug interactions and drug-disease interactions [33, 34]. Rational drug use is therefore critical for all patients.

Strengths of this analysis include the robust and rich data collected on a large sample size of women who were followed for an extended period, which allows the model to identify the trajectories of both variables. In addition, non-HIV medication data was well organized and mapped, making it easy to determine the number of non-HIV medications for each woman at each visit. This study was not without limitations. Firstly, adherence to ART was measured by self-report, which may have been affected by social desirability and recall biases [35]. Secondly, although women were recruited in WIHS from different parts of the country, the obtained results may not be generalizable to all women with HIV. Finally, we cannot rule out the possibility of unmeasured residual confounding.

In conclusion, dual GBTM did not identify an interrelationship between adherence to ART and polypharmacy. Future studies should consider examining the interrelationship between the trajectories of both adherence and polypharmacy using objective measures of adherence to ART.

## Electronic supplementary material

Below is the link to the electronic supplementary material.

Supplementary Material 1

## Data Availability

The data for this analysis were provided by the MACS/WIHS combined cohort study’s Data Analysis and Coordination Center (DACC) with permission. Data will be shared on request to the corresponding author with the permission from the DACC.

## Declarations

### Disclaimer

The contents of this publication are solely the responsibility of the authors and do not represent the official views of the National Institutes of Health (NIH).
