# Supplementary material for: Dual trajectories of antiretroviral therapy adherence and polypharmacy in women with HIV in the United States
Source: AIDS Res Ther. 2023 May 13;20:29. doi: 10.1186/s12981-023-00520-4 (PMC10182649; doi:10.1186/s12981-023-00520-4)
Supplement: Supplementary file 1 — Supplementary Material 1 [file 12981_2023_520_MOESM1_ESM.docx]

**Supplementary file**

Table 1. Characteristics of women in low increasing and moderate decreasing polypharmacy groups at baseline

| Characteristic | Adherence trajectory | | Total  n (%)  428 | P-value |
| --- | --- | --- | --- | --- |
|  | Low increasing  n (%)  N= 160 | Moderate decreasing  n (%)  N= 268 |  |  |
| Age groups  < 50 years  > 50 years | 76 (47.5)  84 (52.5) | 136 (50.7)  132 (49.3) | 212 (49.5)  216 (50.5) | 0.51 |
| Race  White  African American  Hispanic  Others | 11 (6.9)  125 (78.1)  22 (13.7)  2 (1.3) | 31 (11.6)  188 (70.1)  36 (13.4)  13 (4.9) | 42 (9.8)  313 (73.1)  58 (13.5)  15 (3.5) | **0.07** |
| Education  Below secondary  Completed secondary  Some college/ completed college | 60 (37.5)  54 (33.8)  46 (28.7) | 87 (32.5)  78 (29.1)  103 (38.4) | 147 (34.3)  132 (30.8)  149 (34.8) | 0.12 |
| Employment  No  Yes | 115 (72.3)  44 (27.7) | 177 (66.0)  91 (34.0) | 292 (68.4)  135 (31.6) | 0.17 |
| Annual income  = < $ 24,000  >$ 24,000 | 130 (81.2)  30 (18.8) | 208 (77.9)  59 (22.1) | 338 (79.2)  89 (20.8) | 0.41 |
| Alcohol categories  0 drinks/week  >0-7 drinks/week  >7-12 drinks/week  >12 drinks/week | 92 (57.9)  47 (29.6)  8 (5.0)  12 (7.5) | 143 (53.3)  96 (35.8)  15 (5.6)  14 (5.2) | 235 (55.0)  143 (33.5)  23 (5.4)  26 (6.1) | 0.47 |
| History of smoking status  Never smoker  Current smoker  Former smoker | 52 (32.5)  64 (40.0)  44 (27.5) | 89 (33.2)  93 (34.7)  86 (32.1) | 141 (32.9)  157 (36.7)  130 (30.4) | 0.47 |
| Depression symptoms  No  Yes | 103 (64.4)  57 (35.6) | 186 (69.4)  82 (30.6) | 289 (67.5)  139 (32.5) | 0.28 |
| Substance use  No  Yes | 48 (30.0)  112 (70.0) | 70 (26.1)  198 (73.9) | 118 (27.6)  310 (72.4) | 0.38 |

Table 2. The number of groups in the joint model compared to the univariate models

| Number of groups | BIC (N =27303 observations) | BIC (N =1538 participants) | Average BIC |
| --- | --- | --- | --- |
| Adherence = 3 * polypharmacy= 3 | -20988.58 | -20967.01 | 20977.79 |
| Adherence =3 * polypharmacy =4 | -20967.72 | -20940.39 | -20954.05 |
| Adherence =4 * polypharmacy =4 | -20878.59 | -20844.07 | - 20861.33 |
| **Adherence= 5 * polypharmacy= 4** | **-20865.29** | **-20823.58** | **-20844.43** |
| Adherence = 6* polypharmacy = 4 | -20886.56 | -20837.66 | -20862.11 |
| Adherence= 5 * polypharmacy= 5 | 20892.84 | -20842.50 | -20867.65 |

Table 3. Comparing probability of adherence group membership from the univariates and dual models/all medications

| Model | Group membership of Adherence to antiretroviral drugs (%) | | | | |
| --- | --- | --- | --- | --- | --- |
|  | Consistently high | High decreasing | Consistently moderate | Moderate increasing | Consistently low |
| Univariate | 22.1 | 16.3 | 42.3 | 10.5 | 8.8 |
| Dual | 21.2 | 16.1 | 39.9 | 9.0 | 13.8 |

*Sensitivity analysis*

*Trajectories of polypharmacy (considering prescription-only medications)*

GBTM analysis of polypharmacy restricted to prescription-only medications revealed four trajectories namely consistently ‘low’ (N=704; 51.8%), ‘low increasing’ (N=146; 10.8%), ‘moderate decreasing’ (N=170; 12.5%), ‘high increasing’ (N=338; 24.9%) (Figure 3).

Figure (3) Trajectories of polypharmacy/ (considering prescription-only medications)

*Dual trajectory model*

To ensure that the groups identified in univariate GBTM analysis in both adherence and polypharmacy were the best models for the joint analysis, we tested several models by varying the number of groups in each variable. The number of groups identified in the univariate analysis for each variable in the joint model was found to be optimal, as shown in Table 3.

Table 3. The number of groups in the joint model compared to the univariate models /prescription -only medications

| Number of groups | BIC (N = 23259 observations) | BIC (N =1358 participants) |
| --- | --- | --- |
| Adherence =3 * polypharmacy =3 | -18060.30 | -18038.97 |
| Adherence = 3 * polypharmacy = 4 | -18057.81 | -18030.79 |
| Adherence= 4 * polypharmacy= 4 | -17985.91 | -17951.78 |
| **Adherence =5 * polypharmacy= 4** | **-17978.40** | **-17937.17** |
| Adherence= 6 * polypharmacy= 4 | -17998.72 | -17950.37 |
| Adherence= 5* polypharmacy =5 | -18006.53 | 17956.77 |

Table 4. shows a comparison of the probability of group membership of the univariate and joint analysis for both polypharmacy (prescription-only medications) and adherence groups. Except for the consistently low and consistently high adherence groups, there were small changes in the probabilities of group membership in both variables.

Table 4. Comparing the probability of group membership from the univariates and dual models

| Model | Group membership of Adherence to antiretroviral drugs (%) | | | | | | | |
| --- | --- | --- | --- | --- | --- | --- | --- | --- |
|  | Consistently high | High decreasing | | Consistently moderate | | Moderate increasing | | Consistently low |
| Univariate | **21.1** | 15.2 | | 41.5 | | 12.1 | | **10.1** |
| Dual | **16.8** | 14.2 | | 40.7 | | 13.1 | | **15.2** |
|  | Group membership of polypharmacy (%) | | | | | | | |
|  | Consistently high | | Moderate decreasing | | Low increasing | | Consistently low | |
| Univariate | 24.9 | | 12.5 | | 10.8 | | 51.8 | |
| Dual | 25.0 | | 13.0 | | 10.4 | | 51.6 | |

*Interrelationships across the trajectory groups of adherence and polypharmacy (considering prescription-only medications)*

The results of the dual GBTM analysis of polypharmacy groups considering prescription-only medications conditional on adherence groups showed that 51.0% of the women in the consistently low adherence group were members of the consistently low polypharmacy group compared to 50% of the moderate increasing, 50% of the consistently moderate, 48.0% of the high decreasing, and 44.0% of the consistently high as shown in Table 5a. Analysis of adherence groups conditional on polypharmacy groups showed that the consistently moderate adherence group comprised the largest counterparts across all polypharmacy groups as follows: 43.0% of the consistently high, 41.0% of the consistently low, 41.0% of the moderate decreasing, and 30.0% of the low increasing, as shown in Table 5b. The joint probability of adherence and polypharmacy are presented in table 5c. The joint probability of being a member of both the consistently moderate adherence group and the low polypharmacy group was 19.0%, while it was 8% of being a member of both the high decreasing adherence group and the low polypharmacy group.

Table 5a. Probability of polypharmacy group conditional /prescription-only medications on adherence group

| Adherence trajectory group | Polypharmacy trajectory group | | | | Total |
| --- | --- | --- | --- | --- | --- |
|  | Consistently high | Moderate decreasing | Low increasing | Consistently low |  |
| Consistently high | 0.25 | 0.14 | 0.17 | **0.44** | 1 |
| High declining | 0.26 | 0.13 | 0.13 | **0.48** | 1 |
| Moderate increasing | 0.30 | 0.09 | 0.11 | **0.50** | 1 |
| Consistently moderate | 0.26 | 0.15 | 0.09 | **0.50** | 1 |
| Consistently low | 0.13 | 0.21 | 0.15 | **0.51** | 1 |

Table 5b. Probability of adherence conditional on polypharmacy/ /prescription-only medications

| Adherence trajectory group | Polypharmacy trajectory group | | | |
| --- | --- | --- | --- | --- |
|  | Consistently high | Medium decreasing | Low increasing 2 | Consistently low |
| Consistently high | 0.16 | 0.15 | 0.21 | 0.13 |
| High declining | 0.17 | 0.15 | 0.17 | 0.16 |
| Medium increasing | 0.16 | 0.08 | 0.13 | 0.14 |
| Consistently moderate | **0.43** | **0.41** | **0.30** | **0.41** |
| Consistently low | 0.08 | 0.22 | 0.19 | 0.16 |
| Total | 1 | 1 | 1 | 1 |

Table 5c Joint probability of adherence and polypharmacy/prescription-only medications

| Adherence trajectory group | Polypharmacy trajectory group | | | |
| --- | --- | --- | --- | --- |
|  | Consistently high | Medium decreasing | Low increasing | Low |
| Consistently high | 0.04 | 0.02 | 0.03 | 0.07 |
| High decreasing | 0.04 | 0.02 | 0.02 | **0.08** |
| Medium increasing | 0.04 | 0.01 | 0.02 | 0.07 |
| Consistently moderate | **0.11** | 0.06 | 0.04 | **0.19** |
| Consistently low | 0.02 | 0.03 | 0.02 | 0.07 |

*Trajectories of non-HIV medications*

The results of GBTM revealed four trajectories of non-HIV medication namely “consistently very low” (N=504; 32.8%), “consistently low” (N=655; 42.6%), “consistently moderate” (N= 277; 18.0%), and “consistently high” (N=102; 6.6%) Figure 6).

Figure (6): Trajectories of non-HIV medications

*Dual trajectory model*

We tested several models by varying the number of groups in each variable to ensure that the groups identified in univariate GBTM analysis in both adherence and non-HIV medications were the best models for the joint analysis. Table 6 shows that the number of groups identified in the univariate analysis for each variable in the joint model was optimal.

Table 6. The number of groups in the joint model compared to the univariate models

| Number of groups | BIC (N = 27653 observations) | BIC (N =1538 participants) |
| --- | --- | --- |
| Adherence =4 * polypharmacy= 3 | -47859.66 | -47830.78 |
| Adherence = 4 * polypharmacy= 4 | -47264.6 | -47228.52 |
| Adherence= 5 * polypharmacy= 3 | -47841.34 | -47806.69 |
| **Adherence= 5 * polypharmacy= 4** | **-47247** | **-47204.57** |
| Adherence= 6* polypharmacy = 4 | -47271.58 | -47221.05 |

The results showed that there were no significant differences in the probability of group membership when comparing non-HIV in univariate and dual analysis however, there were differences in adherence groups, specifically in the consistently high and consistently low groups, as shown in Table 7.

Table 7. Comparing probability of adherence group membership from the univariates and dual models

| Model | | Group membership of Adherence to antiretroviral drugs (%) | | | | | | | |
| --- | --- | --- | --- | --- | --- | --- | --- | --- | --- |
|  |  | Consistently high | High decreasing | | Consistently moderate | | Moderate increasing | | Consistently low |
| Univariate | | **21.1** | 15.2 | | 41.5 | | 12.1 | | **10.1** |
| Dual | | **15.1** | 17.7 | | 41.3 | | 10.4 | | **15.5** |
|  | Group membership of polypharmacy (%) | | | | | | | | |
|  | High | | | Moderate | | Low | | very low | |
| Univariate | 6.6 | | | 18 | | 42.6 | | 32.8 | |
| Dual | 6.6 | | | 18.3 | | 42.3 | | 32.8 | |

*Interrelationships across the trajectory groups of adherence and non-HIV medications*

Dual GBTM analysis of non-HIV all medications groups based on the number of medications conditional on adherence groups revealed that 52.0% of the women in the high decreasing adherence group were members of the consistently low group compared to 49.0% of the consistently low, 41.0% of the consistently moderate, 36.0% of the consistently high, and 32.0% of the moderate increasing, as shown in Table 8a.

Table 8a. Probability of number of non -HIV medications on adherence groups

| Adherence trajectory group | Non-HIV Medications | | | | |
| --- | --- | --- | --- | --- | --- |
|  | High | Moderate | Low | Very low | Total |
| Consistently high | 0.04 | 0.24 | **0.36** | 0.36 | 1 |
| High decreasing | 0.09 | 0.16 | **0.52** | 0.23 | 1 |
| Consistently moderate | 0.07 | 0.19 | **0.41** | 0.33 | 1 |
| Moderate increasing | 0.09 | 0.21 | **0.32** | 0.38 | 1 |
| Consistently low | 0.03 | 0.13 | **0.49** | 0.36 | 1 |

Analysis of adherence groups conditional on non-HIV groups showed that across all non-HIV groups, the consistently moderate adherence group constituted the largest counterparts as follows: 44% of the consistently high, 41.0% of the consistently moderate, 41% of the consistently very low, and 39.0% of the consistently low increasing group, as shown in Table 8b.

Table 8b. Probability of adherence groups on number of non -HIV medications

| Adherence trajectory group | Non-HIV Medications | | | |
| --- | --- | --- | --- | --- |
|  | High | Moderate | Low | Very low |
| Consistently high | 0.09 | 0.19 | 0.12 | 0.15 |
| High decreasing | 0.25 | 0.16 | 0.23 | 0.13 |
| Consistently moderate | **0.44** | **0.41** | **0.39** | **0.41** |
| Moderate increasing | 0.16 | 0.13 | 0.08 | 0.13 |
| Consistently low | 0.06 | 0.11 | 0.18 | 0.18 |
| Total | 1 | 1 | 1 | 1 |

Table 8c shows the joint probabilities of adherence trajectories and non-HIV medication groups. The joint probability of being a member of both the consistently moderate adherence group and the consistently low non-HIV medication group was 17.0%, while it was 13.0% for being a member of both the consistently moderate adherence group and consistently very low non-HIV medications group.

Table 8c. Joint probability of adherence trajectories and number of non-HIV medications

| Adherence trajectory group | Non-HIV Medications | | | |
| --- | --- | --- | --- | --- |
|  | High | Moderate | Low | Very low |
| Consistently high | 0.006 | 0.03 | 0.05 | 0.05 |
| High decreasing | 0.02 | 0.03 | 0.10 | 0.04 |
| Consistently moderate | 0.03 | 0.08 | **0.17** | **0.13** |
| Moderate increasing | 0.01 | 0.02 | 0.03 | 0.04 |
| Consistently low | 0.004 | 0.02 | 0.08 | 0.06 |
